# Survival without major morbidity in extremely preterm infants: a prospective multicenter study in Shenzhen, China

**DOI:** 10.3389/fped.2026.1859439

**Published:** 2026-07-08

**Authors:** Yanping Guo, Ying Liu, Hua Peng, Songzhou Xu, Xudong Yan, Zhangbin Yu, Guobing Chen

**Affiliations:** 1 Department of Pediatrics, Peking University Shenzhen Hospital, Shenzhen, China; 2 Department of Neonatology, Shenzhen People's Hospital (The Second Clinical Medical College, Jinan University, the First Afffliated Hospital, Southern University of Science and Technology), Shenzhen, China

**Keywords:** antenatal corticosteroids, birth weight, bronchopulmonary dysplasia, extremely preterm infants, multicenter study, survival without major morbidity

## Abstract

**Background:**

Due to regional and economic disparities, the current status of care for extremely preterm infants (EPIs) varies across mainland China and remains incompletely characterized. This study aimed to evaluate the survival rate and major morbidities of EPIs in Shenzhen and to identify factors associated with survival without major morbidity.

**Methods:**

In this prospective multicenter study, clinical data of preterm infants with gestational age (GA) < 28 weeks from 21 hospitals in Shenzhen were collected between January 2022 and December 2023. Baseline characteristics, survival outcomes, and survival without major morbidity were analyzed according to seven GA groups (21–27 weeks). Morbidity analyses among surviving infants were performed using four GA groups (21–24, 25, 26, and 27 weeks). The primary outcome was survival status at discharge or at 40 weeks of postmenstrual age, and the secondary outcomes were the incidences of major morbidities.

**Results:**

A total of 314 EPIs were included, of whom 56 died following withdrawal of care, 24 died despite active treatment, and 234 survived, yielding an overall survival rate of 74.52% (234/314). Among all 314 EPIs, survival without major morbidity was achieved in 24.20% (76/314) of infants. Among the 234 surviving infants, the incidences of major morbidities were as follows: moderate-to-severe bronchopulmonary dysplasia (48.72%, 114/234), severe intraventricular hemorrhage and/or periventricular leukomalacia (13.25%, 31/234), necrotizing enterocolitis stage ≥ 2 (6.41%, 15/234), sepsis (22.65%, 53/234), and severe retinopathy of prematurity (42.31%, 99/234). Multivariable logistic regression analysis showed that antenatal corticosteroid (ACS) use (OR = 7.66), increasing GA (OR = 1.62), and higher birth weight (BW) (OR = 1.77) were independent protective factors for survival without major morbidity.

**Conclusion:**

This prospective multicenter study provides contemporary data on survival without major morbidity among EPIs in Shenzhen. Although overall survival was relatively high, survival without major morbidity remained limited, with substantial burdens of bronchopulmonary dysplasia and retinopathy of prematurity among survivors. ACS exposure, higher GA, and greater BW were significantly associated with survival without major morbidity.

## 1 Introduction

Extremely preterm infants (EPIs), defined as those born at a gestational age (GA) of less than 28 weeks, represent a population at the highest risk of mortality and severe morbidity among preterm infants (1–3). Despite substantial advances in perinatal and neonatal care over recent decades, EPIs remain vulnerable to a wide range of complications, including bronchopulmonary dysplasia (BPD), severe intraventricular hemorrhage (IVH), necrotizing enterocolitis (NEC), sepsis, and retinopathy of prematurity (ROP), all of which may lead to long-term neurodevelopmental impairment and reduced quality of life (4–7).

In high-income countries, continuous improvements in perinatal management strategies—such as the use of antenatal corticosteroid (ACS), optimized respiratory support, and standardized neonatal intensive care—have significantly increased survival rates among EPIs (8, 9). More importantly, increasing attention has been directed toward survival without major morbidity, which is considered a more meaningful outcome reflecting both survival and quality of care (10, 11). However, outcomes of EPIs may vary substantially across regions due to differences in socioeconomic development, healthcare resources, and clinical practices (12).

In mainland China, although neonatal care has improved rapidly in recent years, there remains considerable heterogeneity in the management and outcomes of EPIs across different regions (13). Current evidence is limited, particularly from large-scale prospective multicenter studies, and data regarding survival without major morbidity are still scarce. Shenzhen, as one of the most economically developed cities in China, has relatively advanced perinatal and neonatal care systems, providing a unique opportunity to evaluate contemporary outcomes of EPIs in a well-resourced setting.

Therefore, this prospective multicenter study aimed to investigate the survival rate and incidence of major morbidities among EPIs in Shenzhen, China, and to identify perinatal factors associated with survival without major morbidity.

## 2 Materials and methods

### 2.1 Study design and participants

This multicenter study prospectively collected data from the Shenzhen Neonatal Data Network (SNDN), encompassing preterm infants with GA <28 weeks who received treatment in 21 tertiary neonatal intensive care units (NICUs) across Shenzhen between January 2022 and December 2023. All participating hospitals completed standardized training before the study commenced. Inclusion Criteria: (1) Preterm infants with GA < 28 weeks; (2) admission to the NICU within 24 h of birth. Exclusion Criteria: Infants with major congenital malformations or genetic disorders were excluded.

This study received approval from the Ethics Committee of Shenzhen People's Hospital (Approval No. LL-KY-2022494-02), and other participating institutions agreed. The study protocol has been registered with the Chinese Clinical Trial Registry (ChiCTR2400090262).

### 2.2 Data collection and definitions

Data were retrieved from the electronic medical record systems of each participating institution using a standardized collection form. The following information was recorded: (1) Maternal information: age, gestational hypertension, gestational diabetes, chorioamnionitis (defined based on placental histopathological diagnostic criteria after delivery) (14), occurrence of preterm premature rupture of membranes (PPROM) (defined as rupture of membranes lasting ≥18 h before delivery).(2) Perinatal information: ACS administration, antenatal magnesium sulfate usage, antibiotics usage within 24 h prior to delivery, mode of conception (natural conception or assisted reproduction), multiple births (twins or more), mode of delivery (vaginal delivery or cesarean section), 1-minute Apgar score, 5-minute Apgar score. (3) Neonatal information: sex, GA, birth weight (BW), small for gestational age (SGA), neonatal respiratory distress syndrome (RDS) (15), pulmonary surfactants (PS) usage, early onset sepsis (EOS) (16), late-onset sepsis (LOS) (16), NEC ≥ Bell stage 2 (17), IVH grade 3–4 (18), periventricular leukomalacia (PVL) (18), ROP ≥ stage 3 (19), and BPD (20).

In this study, survivors were defined as neonates who survived to hospital discharge. Neonatal morbidity referred to the occurrence of complications during hospitalization and up to discharge. Gestational diabetes mellitus (GDM) was defined as diabetes occurring during pregnancy, including all types and severities. ACS use was defined as the administration of at least one dose of corticosteroids to the mother via intravenous or intramuscular injection at any time before delivery. A complete course of ACS was defined as the mother receiving four intramuscular injections of 6 mg each at 12-hour intervals, specifically dexamethasone. Severe complications included severe neurological injury, NEC ≥ stage 2) (17), sepsis (16), moderate-to-severe BPD, and severe ROP ≥ stage 3) (19). According to the Papile classification, severe neurological injury was defined as IVH grade 3 or 4, or PVL grade 4 (18). According to the Bell staging criteria, NEC stage 2 or higher was considered morbidity. Sepsis was defined based on a positive blood culture or cerebrospinal fluid culture. BPD was defined as the requirement for oxygen supplementation or ventilatory support at 28 days after birth. Moderate-to-severe BPD was defined as the need for oxygen supplementation or ventilatory support at 36 weeks' postmenstrual age (PMA), or at the time of discharge, transfer, or death before 36 weeks' postmenstrual age (20). Severe ROP was defined as ROP stage 3 or higher, or ROP requiring treatment. Survival without major morbidity was defined as survival without any of the aforementioned severe complications (21).

### 2.3 Outcomes

The primary outcome was survival status at either hospital discharge or 40 weeks' PMA, whichever occurred first. Secondary outcomes included the incidence of major complications. Survival without major morbidity was defined as survival to discharge or 40 weeks' PMA without the occurrence of any of the aforementioned major complications.

### 2.4 Statistical analysis

Infants were stratified by GA for subgroup analyses. Baseline characteristics, survival outcomes, and survival without major morbidity were analyzed using seven GA groups (21–27 weeks). Morbidity analyses were restricted to surviving infants and were performed using four GA groups (21–24, 25, 26, and 27 weeks). The lower GA strata were combined because of the limited number of survivors, thereby improving the stability and interpretability of the estimates.

The normality of quantitative data was assessed using histograms and the Kolmogorov–Smirnov test. Normally distributed variables were expressed as mean ± standard deviation, whereas non-normally distributed variables were summarized as interquartile range. Categorical variables were presented as frequencies and percentages (%).

Univariate analysis was conducted to evaluate the association between perinatal factors and survival without major morbidity. Variables with clinical relevance or those with *P* < 0.10 in univariate analysis were included in a multivariable logistic regression model to identify independent risk factors. Multicollinearity among variables was assessed using the variance inflation factor (VIF), with VIF < 5 indicating no significant multicollinearity. Results were reported as adjusted odds ratios (ORs) with 95% confidence intervals (CIs).

Statistical analyses were conducted using SPSS version 25.0 (IBM, Armonk, NY, USA). A two-sided *P* value < 0.05 was considered statistically significant.

## 3 Results

### 3.1 General characteristics

**Figure 1:**
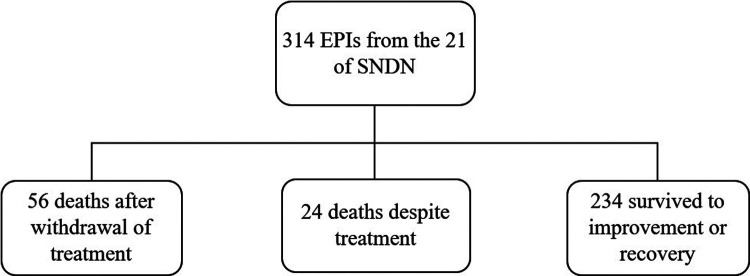
Study flow diagram. Selection of extremely preterm infants included in the analysis from the Shenzhen Neonatal Data Network (SNDN) between January 1, 2022, and December 31, 2023

A total of 314 EPIs were included in this study, of whom 56 died following withdrawal of care, 24 died despite active treatment, and 234 survived to improvement or recovery (Figure 1). Among these 314 EPIs, the median GA was 26.43 (25.29, 27.29) weeks, and the mean BW was 854.22 ± 200.59 g (Table 1). For baseline analyses, the 314 EPIs were stratified into seven GA groups (21, 22, 23, 24, 25, 26, and 27 weeks). Baseline maternal and neonatal characteristics, including gestational hypertension, gestational diabetes mellitus, chorioamnionitis, prolonged rupture of membranes (≥18 h), use of ACS, antenatal magnesium sulfate, antenatal antibiotics, assisted reproductive technology, cesarean delivery, GA, BW, sex, multiple births, 1-minute and 5-minute Apgar scores ≤7, and surfactant administration, were compared across the seven GA groups. Table 1 presents the baseline characteristics of all 314 EPIs stratified by GA.

**Table 1:** Baseline characteristics of 314 extremely preterm infants by gestational age admitted to 21 NICUs in the Shenzhen Neonatal Data Network (SNDN).

Variables	Total (*n*=314)	27 (*n*=112)	26 (*n*=88)	25 (*n*=60)	24 (*n*=33)	23 (*n*=11)	22 (*n*=7)	21(*n*=3)	Statistic	*P*
Age, Mean ± SD	32.02 ± 4.71	31.60 ± 4.75	32.73 ± 4.46	31.70 ± 5.29	31.97 ± 4.29	33.82 ± 5.06	30.57 ± 3.95	30.67 ± 1.15	F=0.94	0.464
GH, *n* (%)	34 (10.83)	20 (17.86)	6 (6.82)	4 (6.67)	2 (6.06)	2 (18.18)	0 (0.00)	0 (0.00)	-	0.122
GDM, *n* (%)	73 (23.25)	30 (26.79)	21 (23.86)	16 (26.67)	6 (18.18)	0 (0.00)	0 (0.00)	0 (0.00)	-	0.281
Chorioamnionitis, *n* (%)	184 (58.60)	62 (55.36)	46 (52.27)	41 (68.33)	22 (66.67)	5 (45.45)	7 (100.00)	1 (33.33)	-	0.050*
PPROM≥18h, *n* (%)	71 (22.61)	26 (23.21)	16 (18.18)	17 (28.33)	9 (27.27)	1 (9.09)	2 (28.57)	0 (0.00)	-	0.634
ACS, *n* (%)	270 (85.99)	98 (87.50)	76 (86.36)	51 (85.00)	31 (93.94)	9 (81.82)	5 (71.43)	0 (0.00)	-	**0.014**
Full course of ACS, *n* (%)	153 (48.73)	57 (50.89)	41 (46.59)	32 (53.33)	20 (60.61)	1 (9.09)	2 (28.57)	0 (0.00)	-	**0.029**
Antenatal magnesium sulfate, *n* (%)	241 (76.75)	87 (77.68)	73 (82.95)	46 (76.67)	23 (69.70)	6 (54.55)	4 (57.14)	2 (66.67)	-	0.195
Antenatal antibiotics, *n* (%)	207 (65.92)	73 (65.18)	53 (60.23)	45 (75.00)	23 (69.70)	5 (45.45)	7 (100.00)	1 (33.33)	-	0.079
Assisted reproduction, *n* (%)	82 (26.11)	30 (26.79)	26 (29.55)	15 (25.00)	7 (21.21)	2 (18.18)	2 (28.57)	0 (0.00)	-	0.946
Vaginal delivery, *n* (%)	171 (54.46)	40 (35.71)	49 (55.68)	38 (63.33)	25 (75.76)	9 (81.82)	7 (100.00)	3 (100.00)	-	<.001*
BW, Mean ± SD	854.22 ± 200.59	978.65 ± 167.22	907.12 ± 132.89	788.40 ± 108.19	652.30 ± 106.24	551.82 ± 98.77	395.71 ± 77.86	373.33 ± 73.71	F=59.23	**<.001**
GA, M (Q₁, Q₃)	26.43 (25.29, 27.29)	27.43 (27.29,27.71)	26.43 (26.29,26.86)	25.43 (25.14,25.71)	24.71 (24.43,24.71)	23.57 (23.29,23.64)	22.14 (22.07,22.43)	21.86 (21.79,21.86)	χ²=289.99#	<.001
Male, *n* (%)	181 (57.64)	60 (53.57)	61 (69.32)	30 (50.00)	16 (48.48)	9 (81.82)	3 (42.86)	2 (66.67)	-	0.051*
Multiple, *n* (%)	152 (48.41)	41 (36.61)	37 (42.05)	32 (53.33)	24 (72.73)	8 (72.73)	7 (100.00)	3 (100.00)	-	<.001*
SGA, *n* (%)	20 (6.37)	6 (5.36)	2 (2.27)	1 (1.67)	2 (6.06)	3 (27.27)	5 (71.43)	1 (33.33)	-	**<.001**
1-minute Apgar score, *n* (%)	141 (44.90)	43 (38.39)	35 (39.77)	30 (50.00)	15 (45.45)	11 (100.00)	5 (71.43)	2 (66.67)	-	<.001*
5-minute Apgar score, *n* (%)	76 (24.20)	16 (14.29)	16 (18.18)	18 (30.00)	9 (27.27)	9 (81.82)	5 (71.43)	3 (100.00)	-	<.001*
PS, *n* (%)	263 (83.76)	86 (76.79)	77 (87.50)	54 (90.00)	27 (81.82)	11 (100.00)	5 (71.43)	3 (100.00)	-	0.118

F, ANOVA; SD, standard deviation; M, median; Q₁, 1st Quartile; Q₃, 3st Quartile; NICU, neonatal intensive care unit; GH, gestational hypertension; GDM, gestational diabetes; PPROM, premature rupture of membranes; ACS, antenatal corticosteroid; BW, birth weight; GA, gestational age; SGA, small for gestational age; PS, pulmonary surfactants.

#: Kruskal-waills test, -: Fisher exact, *: Simulated p-value.

Bold values indicate statistical significance (*P* < 0.05).

### 3.2 Survival and morbidity

The overall survival rate of EPIs was 74.52% (234/314). According to GA subgroups, the survival rates of infants born at 21, 22, 23, 24, 25, 26, and 27 weeks were 0%, 0%, 27.27%, 39.39%, 68.33%, 88.64%, and 88.39%, respectively (Table 2). Among all EPIs, the rate of survival without major morbidity was 24.2% (76/314). For descriptive presentation, the 21–24-week gestational age group showed no survival without major morbidity (0%), and the corresponding rates for 25, 26, and 27 weeks were 13.33%, 27.27%, and 39.25%, respectively (Table 2).

**Table 2:** Survival and survival without major morbidity by gestational age among extremely preterm infants admitted to 21 NICUs.

Variables	Total (*n*=314)	27w (*n*=112)	26w (*n*=88)	25w (*n*=60)	24w (*n*=33)	23w (*n*=11)	22w (*n*=7)	21w (*n*=3)
Survival, *n* (%)^a^	234 (74.52)	99 (88.39)	78 (88.64)	41 (68.33)	13 (39.39)	3 (27.27)	0 (0.00)	0 (0.00)
Survival without major morbidity, *n* (%)^b^	76 (24.20)	44 (39.25)	24 (27.27)	8 (13.33)	0 (0.00)	0 (0.00)	0 (0.00)	0 (0.00)

a Calculated among EPIs admitted to the NICU, including infants discharged against medical advice, those with insufficient information, and those who survived to discharge.

b Calculated using all EPIs included in the study as the denominator (n=314).

Among the 234 surviving EPIs, morbidity analyses were performed according to GA. Because of the limited number of survivors in the lowest gestational-age strata, the 234 survivors were grouped into four GA categories (21–24, 25, 26, and 27 weeks) to ensure stable and interpretable estimates. The overall incidence rates of moderate-to-severe BPD, NEC ≥ stage 2, IVH ≥ grade 3 or PVL, ROP ≥ stage 3, and sepsis were 48.72%, 6.41%, 13.25%, 42.31%, and 22.65%, respectively. The incidence of moderate-to-severe BPD and ROP ≥ stage 3 decreased with increasing GA (Table 3).

**Table 3:** Morbidities by gestational age among surviving extremely preterm infants admitted to 21 NICUs.

Variables	Total (*n*=234)	27w (*n*=99)	26w (*n*=78)	25w (*n*=41)	21-24w (*n*=16)	Statistic	*P*
Moderate-to-severe BPD, *n* (%)	114 (48.72)	32 (32.32)	41 (52.56)	27 (65.85)	14 (87.50)	χ²=25.56	<.001*
NEC stage≥2, *n* (%)	15 (6.41)	6 (6.06)	7 (8.97)	2 (4.88)	0 (0.00)	-	0.718
≥3 grade IVH/PVL, *n* (%)	31 (13.25)	13 (13.13)	9 (11.54)	5 (12.20)	4 (25.00)	χ²=2.16	0.540
Rop stage≥3^a^, *n* (%)	99 (42.31)	30 (30.30)	32 (41.03)	25 (60.98)	12 (75.00)	χ²=18.76	<.001*
Sepsis, *n* (%)	53 (22.65)	14 (14.14)	24 (30.77)	12 (29.27)	3 (18.75)	χ²=8.19	0.042*

χ², Chi-square test; -, Fisher exact; NICU, neonatal intensive care unit; BPD, bronchopulmonary dysplasia; NEC, necrotizing enterocolitis; IVH, intraventricular hemorrhage; PVL, periventricular leukomalacia; ROP, retinopathy of prematurity.

a ROP was evaluated among infants with eye examinations.

*Significant at P< 0.05, as determined by the Fisher test.

### 3.3 Multivariable analysis of survival without major morbidity

Univariate analysis was performed to evaluate the associations between perinatal factors and survival without major morbidity among the full cohort of 314 EPIs. Variables with clinical relevance or with a *P* value <0.10 in the univariate analysis were included in the multivariable logistic regression model to identify independent influencing factors associated with the outcome. Variance inflation factors (VIFs) were used to assess multicollinearity among variables, with VIF <5 indicating no significant multicollinearity.

Multivariable logistic regression analysis showed that antenatal corticosteroid administration (OR = 7.66, 95% CI: 1.93–30.48, *P* = 0.004), increasing GA (per 1-week increase, OR = 1.62, 95% CI: 1.04–2.53, *P* = 0.035), and increasing birth weight (per 100-g increase, OR = 1.77, 95% CI: 1.36–2.31, *P* < 0.001) were independently associated with survival without major morbidity in EPIs (Table 4).

**Table 4:** Multivariable logistic regression analysis for survival without major morbidity.

Variables	β	S.E	Z	*P*	OR (95%CI)
Intercept	-20.87	5.78	-3.61	**<.001**	0.00 (0.00∼0.00)
ACS	2.04	0.70	2.89	**0.004**	7.66 (1.93∼30.48)
Full course of ACS	-0.33	0.33	-1.01	0.314	0.72 (0.38∼1.37)
Vaginal delivery	0.36	0.34	1.06	0.289	1.43 (0.74∼2.79)
GA	0.48	0.23	2.11	**0.035**	1.62 (1.04∼2.53)
Birth Weight Per 100 g	0.57	0.13	4.27	**<.001**	1.77 (1.36∼2.31)
Multiple	-0.41	0.34	-1.22	0.223	0.66 (0.34∼1.29)
SGA	1.56	0.94	1.66	0.098	4.75 (0.75∼30.00)
1-minute Apgar score	-0.37	0.37	-1.00	0.316	0.69 (0.33∼1.42)
5-minute Apgar score	0.27	0.49	0.56	0.574	1.31 (0.51∼3.40)

OR, odds ratio; CI, confidence interval; ACS, antenatal corticosteroid; BW, birth weight; GA, gestational age; SGA, small for gestational age. Data are presented as odds ratios (ORs) with 95% confidence intervals (CIs). Multivariable logistic regression was used to identify independent predictors of survival without major morbidity in 314 extremely preterm infants, including 76 events. Variables with *P* < 0.10 in univariate analyses or clinical relevance were entered into the final model. Multicollinearity was assessed using variance inflation factor (VIF < 5). Model calibration was evaluated using the Hosmer–Lemeshow test (χ²=7.012, P=0.535).

Bold values indicate statistical significance (*P* < 0.05).

To assess the robustness of the results, we further applied a modified Poisson regression model with robust variance estimation and reported adjusted risk ratios (aRRs). The results were consistent with those of the logistic regression analysis (Supplementary Table 1).

Model goodness-of-fit was evaluated using the Hosmer–Lemeshow test (Supplementary Table 2), which indicated a good model fit (*χ*² = 7.012, *P* = 0.535).

The VIF values for all variables ranged from 1.185 to 2.904, indicating no evidence of significant multicollinearity (Table 5).

**Table 5:** Assessment of multicollinearity among covariates included in the multivariable logistic regression model.

Variable	VIF
ACS	1.211
Full course of ACS	1.211
Vaginal delivery	1.206
GA	2.779
Birth weight per 100 g	2.904
Multiple	1.185
SGA	1.308
1-minute Apgar score	1.389
5-minute Apgar score	1.527

VIF, variance inflation factors; ACS, antenatal corticosteroid; BW, birth weight; GA, gestational age; SGA, small for gestational age.

## 4 Discussion

This prospective multicenter study evaluated survival, the burden of major morbidities, and factors associated with survival without major morbidity among EPIs in the Shenzhen. The overall survival rate was 74.52%, whereas only 24.20% of infants survived without major morbidity. Despite the relatively high survival rate, major morbidity remained common among survivors, particularly bronchopulmonary dysplasia and retinopathy of prematurity. These findings provide contemporary multicenter data on the outcomes of EPIs in Shenzhen and highlight the ongoing challenge of improving not only survival but also the quality of survival. Multivariable analysis showed that ACS exposure, higher GA and greater BW were significantly associated with survival without major morbidity.

The overall survival rate observed in this study is comparable to that reported in high-income countries and economically developed regions of China (12, 22), suggesting that neonatal care for EPIs in Shenzhen has achieved a relatively high standard. This progress may reflect ongoing improvements in regional perinatal care systems, advances in neonatal intensive care practices, and the increasing implementation of multidisciplinary care (23). Nevertheless, despite the favorable survival rate, fewer than one-quarter of infants survived without major morbidity, highlighting the ongoing challenge of shifting the focus of clinical care from survival alone to the quality of survival. As a composite outcome, survival without major morbidity captures both survival and the burden of severe complications, thereby providing a more comprehensive assessment of neonatal care quality and clinical outcomes in EPIs.

Notably, the burden of major morbidities among surviving infants remained substantial, with the incidences of BPD and ROP≥3 stage reaching 48.72% and 42.31%, respectively. These rates are broadly consistent with previous reports in cohorts of EPIs (24, 25). Both BPD and ROP are closely associated with extreme organ immaturity, prolonged respiratory support, and oxygen exposure. Their high prevalence highlights that, although considerable progress has been made in improving survival, reducing the burden of chronic pulmonary and visual morbidities remains a major challenge in the care of EPIs. These findings suggest that further optimization of respiratory management and oxygen therapy practices may be needed to reduce the incidence of these complications and improve the quality of survival. Moreover, while the neonatal care network in Shenzhen has achieved encouraging survival outcomes, there remains substantial room for improvement in reducing the burden of major morbidities among survivors.

One of the key findings of this study was that ACS exposure was independently associated with survival without major morbidity. This finding is consistent with extensive previous evidence and further underscores the importance of evidence-based ACS administration in pregnancies at risk of extremely preterm birth (9, 26). ACS has been shown to promote fetal lung maturation, improve postnatal respiratory adaptation, and reduce the risk of several prematurity-related complications. Taken together, these findings suggest that optimizing ACS utilization remains one of the most important modifiable approaches for improving outcomes among EPIs.

In addition, higher GA and greater BW were independently associated with survival without major morbidity. These associations are biologically plausible, as both factors are closely related to organ maturity and physiological reserve (27). Although similar findings have been consistently reported in previous studies, our study provides further evidence from a contemporary prospective multicenter cohort of EPIs in Shenzhen. Furthermore, VIF analysis revealed no significant multicollinearity between GA and BW, suggesting that each contributed relatively independent prognostic information for survival without major morbidity and supporting their value in clinical risk assessment and prediction models.

This study has several strengths. First, the prospective multicenter design, encompassing 21 hospitals across Shenzhen, provides contemporary regional data and enhances the representativeness of the findings. Second, standardized data collection procedures and rigorous quality-control measures ensured data consistency and reliability across participating centers. Third, in addition to evaluating survival, the study assessed survival without major morbidity and the burden of major morbidities, offering a more comprehensive evaluation of clinical outcomes and the quality of neonatal care among EPIs. Furthermore, multivariable analyses were performed to adjust for potential confounders and identify factors independently associated with survival without major morbidity.

However, several limitations should be acknowledged. First, although the study included 21 hospitals across Shenzhen, all participants were recruited from a single metropolitan region; therefore, caution is warranted when extrapolating these findings to areas with different healthcare resources, clinical practices, or population characteristics. Second, several potentially relevant aspects of neonatal care, such as respiratory support strategies, oxygen management, and nutritional interventions, were not available for analysis. Finally, the study was limited to in-hospital outcomes and lacked long-term neurodevelopmental follow-up, which remains an essential component of outcome assessment in EPIs. Future research should incorporate structured longitudinal follow-up to evaluate neurodevelopmental outcomes and provide a more comprehensive assessment of long-term prognosis.

## 5 Conclusions

This prospective multicenter study provides contemporary regional data on survival and survival without major morbidity among EPIs in Shenzhen. Although the overall survival rate was relatively high, survival without major morbidity remained limited, and the burden of major morbidities among survivors remained substantial. ACS exposure, higher GA, and greater BW were independently associated with survival without major morbidity. These findings suggest that, while significant progress has been made in improving survival outcomes, further efforts are needed to reduce major morbidities and enhance the quality of survival among EPIs through continued optimization of perinatal and neonatal care.

## Data Availability

The raw data supporting the conclusions of this article will be made available by the authors, without undue reservation.
