# Supplementary material for: Survival without major morbidity in extremely preterm infants: a prospective multicenter study in Shenzhen, China
Source: Front Pediatr. 2026 Jul 8;14:1859439. doi: 10.3389/fped.2026.1859439 (PMC13388470; doi:10.3389/fped.2026.1859439)
Supplement: Supplementary file 1 [file Table1.docx]

**Table 1.** Baseline Characteristics of 314 Extremely Preterm Infants by Gestational Age Admitted to 21 NICUs in the Shenzhen Neonatal Data Network (SNDN).

| Variables | Total (n = 314) | 27 (n = 112) | 26 (n = 88) | 25 (n = 60) | 24 (n = 33) | 23 (n = 11) | 22 (n = 7) | 21(n = 3) | Statistic | *P* |
| --- | --- | --- | --- | --- | --- | --- | --- | --- | --- | --- |
|  |  |  |  |  |  |  |  |  |  |  |
| Age, Mean ± SD | 32.02 ± 4.71 | 31.60 ± 4.75 | 32.73 ± 4.46 | 31.70 ± 5.29 | 31.97 ± 4.29 | 33.82 ± 5.06 | 30.57 ± 3.95 | 30.67 ± 1.15 | F=0.94 | 0.464 |
| GH, n(%) | 34 (10.83) | 20 (17.86) | 6 (6.82) | 4 (6.67) | 2 (6.06) | 2 (18.18) | 0 (0.00) | 0 (0.00) | - | 0.122 |
| GDM, n(%) | 73 (23.25) | 30 (26.79) | 21 (23.86) | 16 (26.67) | 6 (18.18) | 0 (0.00) | 0 (0.00) | 0 (0.00) | - | 0.281 |
| Chorioamnionitis, n(%) | 184 (58.60) | 62 (55.36) | 46 (52.27) | 41 (68.33) | 22 (66.67) | 5 (45.45) | 7 (100.00) | 1 (33.33) | - | 0.050* |
| PPROM≥18h, n(%) | 71 (22.61) | 26 (23.21) | 16 (18.18) | 17 (28.33) | 9 (27.27) | 1 (9.09) | 2 (28.57) | 0 (0.00) | - | 0.634 |
| ACS, n(%) | 270 (85.99) | 98 (87.50) | 76 (86.36) | 51 (85.00) | 31 (93.94) | 9 (81.82) | 5 (71.43) | 0 (0.00) | - | **0.014** |
| Full course of ACS, n(%) | 153 (48.73) | 57 (50.89) | 41 (46.59) | 32 (53.33) | 20 (60.61) | 1 (9.09) | 2 (28.57) | 0 (0.00) | - | **0.029** |
| Antenatal magnesium sulfate, n(%) | 241 (76.75) | 87 (77.68) | 73 (82.95) | 46 (76.67) | 23 (69.70) | 6 (54.55) | 4 (57.14) | 2 (66.67) | - | 0.195 |
| Antenatal antibiotics, n(%) | 207 (65.92) | 73 (65.18) | 53 (60.23) | 45 (75.00) | 23 (69.70) | 5 (45.45) | 7 (100.00) | 1 (33.33) | - | 0.079 |
| Assisted reproduction, n(%) | 82 (26.11) | 30 (26.79) | 26 (29.55) | 15 (25.00) | 7 (21.21) | 2 (18.18) | 2 (28.57) | 0 (0.00) | - | 0.946 |
| Vaginal delivery, n(%) | 171 (54.46) | 40 (35.71) | 49 (55.68) | 38 (63.33) | 25 (75.76) | 9 (81.82) | 7 (100.00) | 3 (100.00) | - | **<.001*** |
| BW, Mean ± SD | 854.22 ± 200.59 | 978.65 ± 167.22 | 907.12 ± 132.89 | 788.40 ± 108.19 | 652.30 ± 106.24 | 551.82 ± 98.77 | 395.71 ± 77.86 | 373.33 ± 73.71 | F=59.23 | **<.001** |
| GA, M (Q₁, Q₃) | 26.43 (25.29, 27.29) | 27.43 (27.29,27.71) | 26.43 (26.29,26.86) | 25.43 (25.14,25.71) | 24.71 (24.43,24.71) | 23.57 (23.29,23.64) | 22.14 (22.07,22.43) | 21.86 (21.79,21.86) | χ²=289.99# | <.001 |
| Male, n(%) | 181 (57.64) | 60 (53.57) | 61 (69.32) | 30 (50.00) | 16 (48.48) | 9 (81.82) | 3 (42.86) | 2 (66.67) | - | 0.051* |
| Multiple, n(%) | 152 (48.41) | 41 (36.61) | 37 (42.05) | 32 (53.33) | 24 (72.73) | 8 (72.73) | 7 (100.00) | 3 (100.00) | - | **<.001*** |
| SGA, n(%) | 20 (6.37) | 6 (5.36) | 2 (2.27) | 1 (1.67) | 2 (6.06) | 3 (27.27) | 5 (71.43) | 1 (33.33) | - | **<.001** |
| 1-minute Apgar score, n(%) | 141 (44.90) | 43 (38.39) | 35 (39.77) | 30 (50.00) | 15 (45.45) | 11 (100.00) | 5 (71.43) | 2 (66.67) | - | **<.001*** |
| 5-minute Apgar score, n(%) | 76 (24.20) | 16 (14.29) | 16 (18.18) | 18 (30.00) | 9 (27.27) | 9 (81.82) | 5 (71.43) | 3 (100.00) | - | **<.001*** |
| PS, n(%) | 263 (83.76) | 86 (76.79) | 77 (87.50) | 54 (90.00) | 27 (81.82) | 11 (100.00) | 5 (71.43) | 3 (100.00) | - | 0.118 |
| Notes: F: ANOVA, #: Kruskal-waills test, -: Fisher exact, *: Simulated p-value, SD: standard deviation, M: Median, Q₁: 1st Quartile, Q₃: 3st Quartile, NICU: neonatal intensive care unit, GH: Gestational Hypertension, GDM: gestational diabetes, PPROM: premature rupture of membranes, ACS: antenatal corticosteroid, BW: birth weight, GA: gestational age, SGA: Small for Gestational Age, PS: pulmonary surfactants. | | | | | | | | | | |
