# Supplementary material for: Survival without major morbidity in extremely preterm infants: a prospective multicenter study in Shenzhen, China
Source: Front Pediatr. 2026 Jul 8;14:1859439. doi: 10.3389/fped.2026.1859439 (PMC13388470; doi:10.3389/fped.2026.1859439)
Supplement: Supplementary file 2 [file Table2.docx]

**Table 2.** Survival and Survival Without Major Morbidity by Gestational Age Among Extremely Preterm Infants Admitted to 21 NICUs.

| Variables | Total (n = 314) | 27w (n = 112) | 26w (n = 88) | 25w (n = 60) | 24w (n = 33) | 23w (n = 11) | 22w (n = 7) | 21w (n = 3) |
| --- | --- | --- | --- | --- | --- | --- | --- | --- |
|  |  |  |  |  |  |  |  |  |
| Survival, n (%) ^a^ | 234 (74.52) | 99 (88.39) | 78 (88.64) | 41 (68.33) | 13 (39.39) | 3 (27.27) | 0 (0.00) | 0 (0.00) |
| Survival without major morbidity, n (%) ^b^ | 76 (24.20) | 44 (39.25) | 24 (27.27) | 8 (13.33) | 0 (0.00) | 0 (0.00) | 0 (0.00) | 0 (0.00) |
| ^a^ Calculated among EPIs admitted to the NICU, including infants discharged against medical advice, those with insufficient information, and those who survived to discharge.  ^b^ Calculated using all EPIs included in the study as the denominator (n = 314). | | | | | | | | |
